# Effect on Feeding Behaviour and Growing of Being a Dominant or Subordinate Growing Pig and Its Relationship with the Faecal Microbiota

**DOI:** 10.3390/ani14131906

**Published:** 2024-06-27

**Authors:** Juan Ochoteco-Asensio, Gustavo Zigovski, Leandro Batista Costa, Raquel Rio-López, Adrià Clavell-Sansalvador, Yuliaxis Ramayo-Caldas, Antoni Dalmau

**Affiliations:** 1 Animal Welfare Program and Animal Breeding and Genetics Program, Institute of Agrifood and Technology (IRTA), Veïnat de Sies s/n, 17121 Monells, Spain; juan.ochoteco@irta.cat (J.O.-A.); raquel.rio@irta.cat (R.R.-L.); adria.clavell@irta.cat (A.C.-S.); yuliaxis.ramayo@irta.cat (Y.R.-C.); 2 Graduate Program of Animal Science, Pontificia Universidade Católica do Paraná-PUCPR, Curitibia 80215-901, Paraná, Brazil; gustavozipaula@gmail.com (G.Z.); batista.leandro@pucpr.br (L.B.C.)

**Keywords:** animal behaviour, animal welfare, dominance, gut microbiota, subordinate, swine

## Abstract

**Simple Summary:**

The present study assessed the effect of being dominant or subordinate within a group of pigs on performance (growth level), feeding behaviour (how many times the animals were visiting the feeder during the day and for how long), and faecal microbiota (the predominant genus of bacteria found in the faeces of pigs along the study). To do it, 64 pigs were studied during their growing period, beginning at two months old with 18 kg and finishing at 7 months old with 140 kg. In the meantime, they were weighed five times, faecal samples were taken from the animals three times, and a hierarchical test to determine the most dominant and subordinate pig in each pen was also repeated three times. The results showed that most pigs, 75%, experienced changes in their social status during the growing period. In addition, the dominant animals had a higher weight at the end of the study, indicating accelerated growth, in addition to visiting the feeder more frequently than the subordinates. Moreover, bacterial genera were associated with either dominant or subordinate animals, indicating a link between social status and some biomarkers in the faeces.

**Abstract:**

Pigs are a social species, and they establish hierarchies for better use of resources and to reduce conflicts. However, in pig production, the opportunities for growth can differ between dominant and subordinate animals. In the present study, a system was tested to perform a dominant versus subordinate test in growing pigs to investigate how the hierarchy affects feeding behaviour, growth, and gut microbiota assessed in faeces. Sixty-four animals housed in eight different pens were used, with four castrated males and four females in each one, weighing 18 kg at arrival and maintained during the whole growing period, until 140 kg. Three stool samples were obtained from the animals directly from the anus to avoid contamination of the faeces 58, 100, and 133 days after the start of the study to investigate the microbiota composition. The dominant animals had higher gains during the growing period than the subordinates. In addition, they were performing more visits to the feeder throughout the day. Differential abundance patterns were observed in five bacterial genera, with *Oliverpabstia*, *Peptococcus*, and *Faecalbacterium* being more abundant in dominant animals and *Holdemanella* and *Acetitomaculum* being overrepresented in subordinate ones. This microbial biomarker accurately classified dominant versus subordinate groups of samples with an AUC of 0.92.

## 1. Introduction

One of the most significant challenges in contemporary pig production is to improve efficiency and reduce housing costs without compromising animal welfare or farm sustainability [1]. Pigs are a social species, and they establish hierarchies for better use of resources and to reduce conflicts. This is not different in commercial conditions, where it is common for pigs to form hierarchical groups where some animals hold a dominant position over others [2]. Although being a dominant or subordinate animal depends on several factors, such as temperament and genetics, one of the most important is body weight and age, as older and larger pigs have more opportunities to win a fight. Consequently, although there are exceptions, there is a high correlation between the size of the pigs and the probability of being a dominant animal in the pen. Larger pigs have the privilege of eating in their preferred manner, while smaller pigs face difficulty accessing feeders, which are often occupied by high-ranking animals [3] in the preferred hours of the day. If the animals are mixed with unknown individuals after the group has been created, they will establish new hierarchies, leading to aggressive behaviour and fighting [4].

In any case, cohorts of pigs will be formed by dominant and subordinate animals, and the fact that these hierarchies will be more or less important will depend on the management done by the farmer. For instance, if the animals are mixed regularly, the formation of hierarchies will be very visible in the form of fights and injuries produced by the animals to each other, and if there is a high level of competition for resources, the difference between the dominant and subordinate animals will also be more evident. In this respect, one of the most important factors of competition in a pig farm is space, so stocking density will be a key factor to give importance to the hierarchy. In fact, a high population density of pigs in a pen has a negative impact on their comfort, whereas a lower pen density results in improvements in animal welfare indicators [5]. Therefore, both the hierarchy formation after mixing unknown animals and stocking density are factors that affect pig production, especially in terms of welfare and zootechnical parameters [6,7].

The present study assumes then that dominant and subordinate pigs reared under two scenarios, one with limited mixing of animals and low stocking density in comparison with one with two mixing procedures during the growing period and higher stocking density, will have different patterns of feeding when registered with automatic feeders. However, in addition to this, although all pigs will be fed in the same environment and group size and with the same diet, the present study hypothesises that the gut microbiota present in both types of animals (dominant and subordinate) will differ. It is known that there is a link between gut microbiota and growth rates^8^, that there is a shift in the gut microbial communities as pigs age [8], and that intestinal health is an important factor for maintaining animal health and performance [9]. However, research assessing the intestinal microbiota and its relationship with pig hierarchies and behaviour is in its early stages. Therefore, studies that interweave the gut microbiota with hierarchical positions and different stocking densities and the possibility of identifying microbial biomarkers of such conditions are of relevance at this moment.

To determine the arrangement of hierarchies, it is necessary to observe the interactions between the animals [4,10]. Therefore, in the present study, a dominance versus subordinate test will be subjected to growing pigs, including a control treatment (no mixing and low stocking densities) and a stress treatment (mixed twice during the growing period and with a higher stocking density). The hypothesis of the study is that the social status (dominant vs. subordinate) within a group of pigs substantially affects their performance (growth level), feeding behaviour (frequency and duration of feeder visits), and faecal microbiota composition (predominant bacterial genera), where external stressors may play a role in exacerbating those differences. The objectives of the study include determining the impact of social stress on hierarchy formation, evaluating the effects of hierarchy on feeding behaviour, assessing the influence of hierarchy on growth, and investigating the relationship between hierarchy and faecal microbiota composition. By achieving these objectives, the study aims to provide a comprehensive understanding of how (stress-induced) hierarchical structures among growing pigs affect their overall well-being, specifically focusing on feeding behaviour, growth rates, and gut microbiota.

## 2. Materials and Methods

### 2.1. Animals and Treatments

This study was carried out on an experimental farm for growing pigs in Monells, Girona, Spain, from June to October 2022. Eight pens were used for this study, with eight piglets of Duroc breed in each pen, four castrated males and four females (64 animals in total), weighing 18 kg (with a mean of 18.8 kg +/− 1.78 SD for females and 18.6 kg +/− 1.64 for males) at arrival (2 months old) and maintained during the whole growing period until 140 kg (7 months old). Four of those pens were considered the stress treatment, which consisted of a higher density (1 m^2^ per animal) and mixing animals twice during the growing period. The other 4 pens were considered the control treatment, which consisted of a lower density (1.5 m^2^ per animal) and the maintenance of stable groups during the growing period. The first mixing of animals for the stress treatment, which consisted of moving just the females among pens, was performed 61 days after the start of the study, and the second one, which consisted of moving just the males among pens, was performed 22 days later. In both cases, for males and females, three animals were changed to the other three pens (one per pen), and the fourth, the smallest female and the smallest male, remained in the original pen. This study was divided into three phases. Phase 1 was defined as the period prior to any mixture, phase 2 as the period after the first mixture (female pigs) and before the second mixture (male pigs), and period 3 as the period after the second mixture until slaughter.

### 2.2. Dominance versus Subordinate Test

To study the relationship between animals in terms of dominance and subordination, all the possible combinations of two individuals in each pen of 8 pigs were tested, amounting to a total of 28 distinct combinations per pen. The test was based on an adaptation of Parent et al. (2012) [11] and consisted of different consecutive sessions over 7 days in a way that each animal did the test only once per day (4 pairing pigs per pen and day). The full process was repeated in three instances during the growing period: just before the first mixing of the stress treatment animals, just after the first mixing, and just after the second mixing. The procedure entailed placing two pigs at the entrance of an outdoor testing pen (4 m long and 4 m wide) that contained a trough with sliced apples just on the opposite side (around 100 g was offered each time). The pigs underwent prior training, during which the group was acclimated to the testing pen, and they were accustomed to consuming apples from the trough. The initial assessment aimed to ascertain which animal initiated the consumption of apples and whether any territorial displacements occurred during the test. In cases where no clear displacement occurred, the animal that first ate the apple was deemed the dominant one (“Dominant”). In the event of displacement or confrontation, dominance status was set for the winner. Subsequently, following no more than 30 s of apple consumption, the dominant animal was gently removed from the feeding area and relocated to the pig’s entrance to facilitate the evaluation of the second pig. This second pig was then granted one minute of solitary access to the apples, after which the dominant pig was reintroduced. A draw was recorded if the subordinate pig consumed the apples without intervention by the dominant counterpart. The dominance status was confirmed if the dominant pig successfully displaced the subordinate during this phase (“Dominant Confirmed”). This second phase allotted one minute for the dominant pig to assert its dominance. After this interval, the test was concluded. The test was deemed inconclusive and non-evaluable in instances where neither of the two pigs consumed apples within a two-minute window or if an animal considered dominant the first time was not able to displace the other pig the second time.

To quantify the dominance test results, two measurements were used: “Dominant” (first animal eating in the first assessment or winner of a fight) and “Dominant Confirmed” (first animal eating in the first assessment and animal displacing the other pig in the second one). If a dominant or confirmed dominant were annotated, it was quantified as a positive qualification in their hierarchy (+1), while the other animal that intervened was quantified negatively (−0.5). In the case of a draw, the contribution to their hierarchy was neutral (0). For a specific time range (before or after a mix), the mean for both dominant and confirmed dominant was calculated for each animal. After that, a ranking score was built as follows: $Ranking\;score=\frac{Dominant}{2}+Dominant\;Confirmed$. A higher-ranking score indicated a higher position in the dominance hierarchy. Thus, we established the two highest-ranking animals per pen as dominant, the two lowest as submissive, and the rest (4) as intermediate. As already mentioned, we repeated the test three times throughout the study: just before the stress treatment pigs were mixed, just after the first/female mixture, and just after the second/male one.

### 2.3. Feeding Behaviour

The animals were identified using ear electronic tags (e-tags), which provided information on the daily consumption of feed in terms of quantity (expressed as the total mass in grammes) and duration. These data were acquired using an electronic feeding system. Several measurements were derived from this dataset:

1. Feed intake: This metric quantified the total mass, in grammes, consumed by each animal per day.
2. Number of feeder visits per day: This measurement indicated how frequently each animal visited the electronic feeder daily.
3. Total feeding duration per day: This metric represented the cumulative time during which each e-tag was in contact with the electronic feeder within a single day.
4. Median duration of feeding session: This measurement reflected the median duration of all feeding sessions from each day for each animal, from which the median of medians was taken.
5. Time slots: This category included the total feeding duration within a 4 h time window, measured both in absolute terms (seconds) and as a percentage of the total feeding time in a day.

Additionally, these measurements were normalised by dividing the value obtained by each pig individually by the median value of all the pigs in their respective pen. This standardisation allowed for the assessment of changes relative to other animals sharing the same pen, independent of the passage of time. As food consumption depends on the size and age of the animals, this effect was controlled throughout the study thanks to standardisation. In addition, weight measurements were recorded for each animal on five occasions: at 0 (beginning), 21, 77, 103, and 134 days from the beginning of the study. The final weight measurement was also conducted at 141 or 148 days, depending on the sacrifice date, when the animals were 7 months old, as 50% of the animals were slaughtered on day 141 and the other 50% on day 148 of the study.

### 2.4. Microbial DNA Extraction, Sequencing, and Bioinformatics Analysis

Three stool samples were obtained from the animals directly by stimulation to facilitate their release and were collected directly from the anus before they contacted the ground to avoid contamination of the faeces 58, 100, and 133 days after the start of the study to investigate the microbiota population. Microbial DNA was extracted with the DNeasy PowerSoil Kit (QIAGEN, Hilden, Germany) following the manufacturer’s recommendations. Extracted DNA was sent to the Centro de Regulación Genómica (CRG) for paired-end (2 × 300 bp) sequencing on an Illumina MiSeq (Illumina, San Diego, CA, USA). The 16S rRNA gene fragment was amplified using the primers V3_F357_N: 5′-CCTACGGGNGGCWGCAG-3′ and V4_R805: 5′-GACTACHVGGGTATCTAATCC-3′. Sequences were analysed with QIIME2 [12]; barcode sequences, primers, and low-quality reads (Phred score < 30) were removed. The quality control process also trimmed sequences based on the expected amplicon length and removed chimeras. Afterward, sequences were clustered into Amplicon Sequence Variants (ASVs) at 99% identity. ASVs were classified to the lowest possible taxonomic level based on a primer-specific trained version of the GreenGenes2 Database (released in October 2022) [13]. Before estimating the diversity indices, samples were rarefied at 31,731 reads to correct for the sequencing depth. Diversity metrics were estimated with the vegan R package v2.6-2 [14]. The α-diversity was evaluated with the Shannon index [15], and the β-diversity was assessed using the Whittaker index [16].

### 2.5. Statistics

All statistical analyses were conducted using R version 4.3.1 (16 June 2023 ucrt) [17], with a significance threshold set at *p* < 0.05. Below, we detail the specific statistical methods employed for each analysis.

#### 2.5.1. Weight versus Hierarchy

The weight measurements of each animal were collected and integrated with their respective hierarchical status within the designated experimental phase. In Phase 1, weight measurements were conducted on two occasions. In the subsequent phase, weight assessments were limited to a single instance. During the third and final phase, most of the animals underwent tripartite weight measurements.

When only a single weight measurement was available for a given phase, a linear regression model was constructed. In this model, the dependent variable was the weight of the animal, and the independent variable was the hierarchical status, treated as a categorical factor. Additionally, treatment and age were introduced as covariates in recognition of their known influence on weight. The reference category for hierarchy was “Dominant” (in contrast with “Intermediate” and “Submissive”), while the reference category for treatment was “Control” (in contrast with “Stress”). The *p*-values derived from a linear model were based on the t-tests for each coefficient.

Conversely, in instances where multiple weight measurements were obtained, a mixed-effects linear model was employed. This mixed-effects model retained the same structure as the simple linear model, encompassing weight as the dependent variable, hierarchical status as a categorical factor, and the covariates of treatment and age. However, it introduced a random effect component attributed to each animal’s unique identification (ID). The lmerTest package was used to enhance the lmer function, which built the mixed-effects models, by providing *p*-values for the fixed effects. The *p*-values were derived from *t*-tests, which used Satterthwaite’s approximation.

#### 2.5.2. Feeding Behaviour versus Hierarchy

In the context of the analysis of feeding behaviour measurements, for each experimental phase and animal, median values were computed (except for time slots, where means were used). Subsequently, linear models were constructed, utilising these values as the dependent variable, with hierarchical ranks (dominant and submissive) as independent categorical factors and treatment and pen as covariate factors. Pen was excluded as a covariate when the dependent variable consisted of relative measurements (normalised by the median value within the respective pen). Similar to weight analysis, significance testing relied on t-tests derived from linear model coefficients.

Changes in hierarchical classes across the experiment were classified as “Better” if the change implied a hierarchical improvement (submissive to either intermediate or dominant, intermediate to dominant), and “Worse” if the change worsened their hierarchical position (dominant to either intermediate or submissive, intermediate to submissive). The linear mixed model was composed of feeding behaviour as the dependent variable; the fixed effect was the interaction between the phase factor (reference being the earliest phase) and the hierarchical change factor (reference being “Better"); and the random effect was the animal’s ID.

In the analysis of the relationship between feeding behaviour and hierarchy (or its change), two animals were excluded from the study due to incomplete feed data, although their hierarchy status was recorded for all instances (except for one of them in phase 3, and therefore it was used for the standalone phase 3 analysis). The primary consequence of this exclusion was a statistical comparison of 16 dominant animals with 15 submissive animals in the third phase of the study, in addition to the exclusion when their hierarchical classes changed: two animals less in the worsened group from phase 2 to phase 3 (19 versus 17), and an animal less in the worsened group from phase 1 to phase 3 (15 versus 14).

#### 2.5.3. Hierarchy Class Changes

In order to evaluate whether the frequency of specific hierarchical changes was statistically different across treatments or compared with what would be expected at random, Pearson’s Chi-squared test for count data was used.

#### 2.5.4. Microbial Biomarker Identification

Biomarker identification was performed with NetMoss2 [18], an R-based tool developed for integrating large-scale datasets to identify microbial biomarkers. NetMoss2 is based on a network-based differential abundance approach to identify driver bacteria associated with state transitions. Therefore, allowing the identification of significant biomarkers in the transition between two conditions, like in our case, dominant vs. submissive pigs, a microbial co-occurrence network was constructed using SparCC [19] based on the raw genus abundance matrix of each condition. Network graphical representation was performed with CytoScape [20]. The topological parameters of the network and nodes’ centralities were calculated using the CentiScaPe plugin. The module division was calculated using WGCNA [21], and the NetMoss score that is used to measure the driving force of every node in the transition of the network structure was estimated as described in [18] using the following model:

$${NMSS\left(i\right)}_{A\to B}=\sum_{j}^{NeighborsA}∆D_{ij}-\sum_{l}^{NeighborsB}∆D_{il}$$

where *A* and *B* represent the dominant and subordinate networks, respectively. Neighbors*A* represents all neighbouring modules in the dominant network, and Neighbors*B* represents all neighbouring modules in the subordinate network. The FDR method was employed to correct for multiple testing, and the classification performance of the microbial biomarkers was evaluated after 5-fold cross-validation as implemented by the ‘*netROC*’ function of NetMoss2.

## 3. Results

In the study, several parameters were compared between dominant and submissive pigs across different phases. The results demonstrate significant differences in weight, food consumption, feeding behaviors, and microbial biomarkers between these two groups. Table 1 provides a comprehensive summary of these comparisons, highlighting the statistical significance of each observed difference.

### 3.1. Effect of Hierarchy on Weight

**Figure 1:**
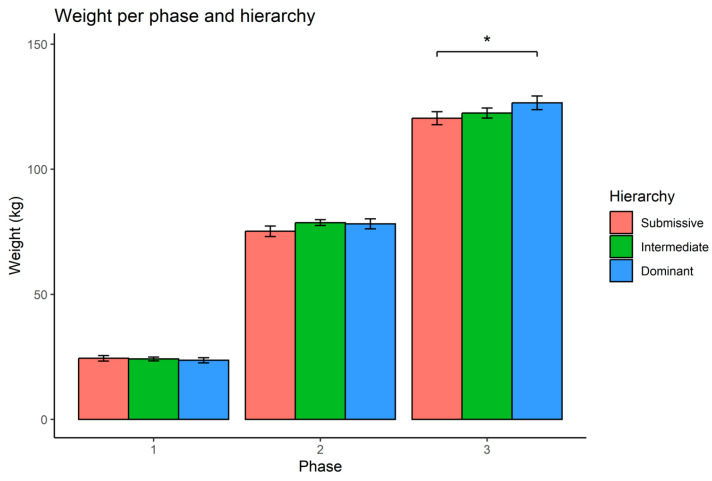
Mean weight (kg) per phase and hierarchy class with standard deviation error bars. A statistically significant difference between submissive and dominant classes in phase three (*p* = 0.018) is indicated by an asterisk (*). No significant difference was observed between Intermediate and Dominant classes.

The results showed that differences between the weights of dominant and submissive animals increased across the experimental phases. In the third phase, a statistically significant difference between these two hierarchical categories emerged (*p*-value = 0.018) (Figure 1), with dominant animals exhibiting greater body weight than their submissive counterparts.

### 3.2. Hierarchy and Feeding Behaviour

**Figure 2:**
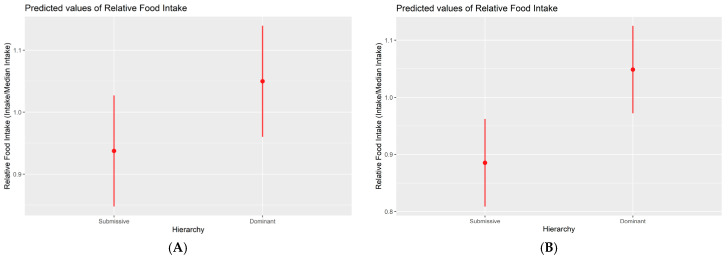
Marginal effects plot between relative food intake (at pen level) and hierarchy (phase 2 (**A**) and phase 3 (**B**)). Higher hierarchy classes correspond to increased food consumption. The difference between submissive and dominant classes is statistically significant in both cases (*p* < 0.05).

Upon thorough examination of various feeding parameters, including total intake, feed rate, daily time spent eating, median time spent per visit, the number of visits, and time spent patterns across different periods, no statistically significant differences were discernible among the three hierarchical classes during the initial experimental phase. During the second phase, discernible alterations in feeding behaviour materialised for the first time between submissive and dominant animals. Submissive pigs exhibited a lower level of food consumption (mean of 2476 +/− 97.7 g) compared with their dominant counterparts (mean of 2745 +/− 118 g). Although the *p*-value is 0.049, indicating statistical significance, this result is at the borderline and should be interpreted with caution. Similar patterns emerged when employing relative measures based on the pen’s median. Submissive animals exhibited 11% lower food consumption compared with dominants (*p*-value = 0.034), as illustrated in Figure 2. In the third and final phase, submissive pigs continued to exhibit a significantly lower food consumption level (mean of 2870 +/− 104 g) compared with their dominant counterparts (mean of 3387 g +/− 93.6, *p*-value = 0.002). This trend persisted when employing relative measures based on the pen’s median, with submissive animals displaying an 18% lower food consumption than dominants (*p*-value = 0.001; Figure 2).

**Figure 3:**
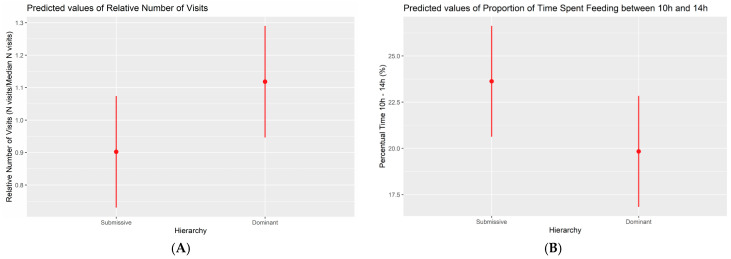
Marginal effects plots for phase 3: (**A**) number of visits and (**B**) percentage of time spent eating (10:00 h–13:59 h). Significant differences between dominant and submissive pigs are observed in all plots.

The frequency of visits to the electronic feeder exhibited variations among hierarchical classes. Specifically, submissives displayed a 22% reduction in visits (Figure 3) relative to the pen’s median, with dominants serving as the reference category (*p*-value = 0.034). Another significant alteration observed in phase 3 pertained to the daily total duration of feeding visits. In relative terms, submissive animals exhibited a 12% decrease (*p*-value = 0.041) in daily feeding duration compared with dominants. For the last significant change between both hierarchical classes, submissives spent proportionally more time feeding between 10 h and 13:59 h than dominants, both across all pens (3.80% increase, *p*-value = 0.033, Figure 3), and relative to each pen (17% increase, *p*-value = 0.030). Finally, although there was an observed numerical difference of 58 s more in median time per meal for submissive pigs compared with dominant subjects, this difference was not statistically significant (*p* = 0.427).

### 3.3. Changes in Hierarchical Status

Across the duration of the experiment, a substantial proportion of the animals experienced alterations in their hierarchical classes, and these changes were not limited to the pigs that were relocated to a different pen (stress group). In global, most animals did change their hierarchical class at some point throughout the experiment: 16 out of the 64 animals remained in their class for the whole study. The proportion of these 16 stable animals differed for each class: 37.5% for dominants (37.5% for both Control and Stress groups), 25% for Intermediate (18.8% for Control and 31.3% for Stress), and 12.5% for submissives (25% for Control and 0% for Stress). No significant differences (*p*-value > 0.05) were found between any of the groups (hierarchy) or subgroups (hierarchy-treatment).

From phase 1 to phase 2, the following changes were observed: out of the 16 dominant animals in phase 1, five of them changed to intermediate (three from the Control group and two from the Stress group)**,** and one to submissive (Stress); out of the 32 intermediate animals in phase 1, five became dominant (three Control, two Stressed), and eight became submissive (five from Control, three from Stress); and out of the 16 submissive animals, one became dominant (Stress group), and eight became intermediate (five Control, three Stressed). Interestingly, all changes were exactly balanced out by their opposite transitions. No statistically significant changes were found in any behavioural indicators between animals that climbed (14, eight Control and six Stress) or descended (14, eight Control and six Stressed) the hierarchical ladder.

**Figure 4:**
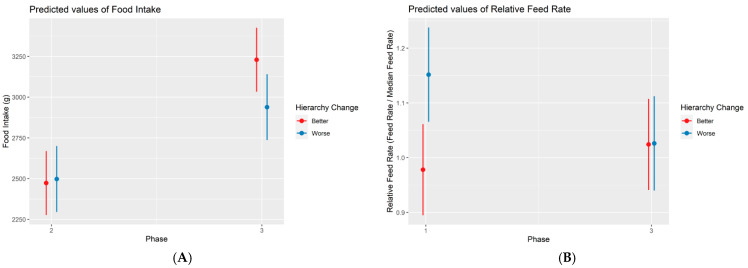
Marginal effects plot illustrating the association between food intake and hierarchy change between phases 2 and 3 (**A**). A marginal effects plot illustrating the association between the food intake and hierarchy change between phases 2 and 3. The interaction between phase 3 and a worsened hierarchical change was found to present a significant negative effect (*p* < 0.05). (**B**). A marginal effects plot illustrating the association between the relative feed rate and hierarchy change between phases 1 and 3. The interaction between phase 3 and a worsened hierarchical change was found to present a significant negative effect (*p* < 0.05).

From phase 2 to phase 3, the following changes were observed: out of the 16 dominant animals in phase 2, six of them changed to intermediate (four Control and two Stressed), and one changed to submissive (Stress); out of the 31 intermediate animals in phase 2, seven became dominant (four Control and three Stressed), and 10 became submissive (four Control and six Stressed); and out of the 16 submissive animals, 12 became intermediate (five Control and seven Stressed). When comparing statistically the animals whose hierarchical status improved (19, nine Control and 10 Stressed) and worsened (17, eight Control and nine Stressed), the total daily intake was found to be decreased (−315.78 g on average) for the worsened animals (Figure 4) in phase 3 (*p*-value = 0.034).

From phase 1 to phase 3, the following changes were observed: out of the 16 dominant animals in phase 1, seven of them changed to intermediate (four Control and three Stressed), and one to submissive (Stressed); out of the 31 intermediate animals in phase 1, seven became dominant (four Control and three Stressed), and six became submissive (two Control and four Stressed); and out of the 16 submissive animals, one became dominant (Stressed), and seven became submissive (three Control and four Stressed). When comparing statistically the animals that improved (15, seven Control and eight Stressed) and worsened (14, six Control and eight Stressed), the relative feed rate was found to be decreased (−17% on average) for worsened animals (Figure 4) in phase 3 (*p*-value = 0.047).

#### Extreme Examples

Among all the hierarchical changes, only two animals, F840 and M917, exhibited an extreme transition that persisted over time. At the beginning of the experiment, F840 held a dominant status, while M917 occupied a submissive role. Initially, both animals belonged to the same pen. During the initial mixing (from phase 1 to phase 2), F840 was transferred to a different pen, where it transitioned from a dominant to a submissive position (remaining submissive in phase 3). Conversely, M917, which remained in its original pen, transitioned from submissive to dominant (from phases 1 to 2) and maintained this status in phase 3. Given these extreme changes, these two cases were individually scrutinised to determine whether the observed shifts in hierarchy were mirrored by alterations in feeding behaviour.

**Figure 5:**
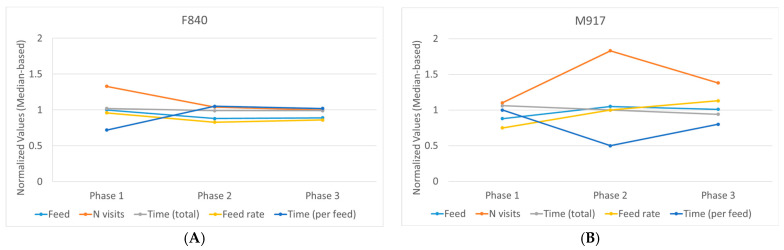
Normalised values for each of the behaviour measurements for both F840 (**A**) and M917 (**B**) across all phases. The values were normalised by dividing the original values by the median of their corresponding pens.

For F840, intake, feed rate, and especially the number of visits decreased along with its hierarchical status compared with its (Pen) peers (Figure 5A). On the other hand, the median duration of its visits increased (similar to the non-significant difference found at the group level). The total time, though, did not change. Globally, the altered levels in phase 2 were kept in phase 3. In the case of M917, an inverse effect was observed. Feed, feed rate, and especially the number of visits increased along with its hierarchical status compared with its (Pen) peers (Figure 5B). On the other hand, the median duration of its visits decreased. As with F840, the total time did not change substantially, although a slight decrease was observed over time. The changes in phase 2 were in part balanced out in phase 3, but the changes were nonetheless still present.

### 3.4. Microbiota Characterization

With faecal samples from all animals in phase 3, their microbiota was sequenced and quantified. From there, the analysis was focused on contrasting the diversity indices and genus abundance between the dominant and submissive hierarchical groups of samples.

#### 3.4.1. Diversity

**Figure 6:**
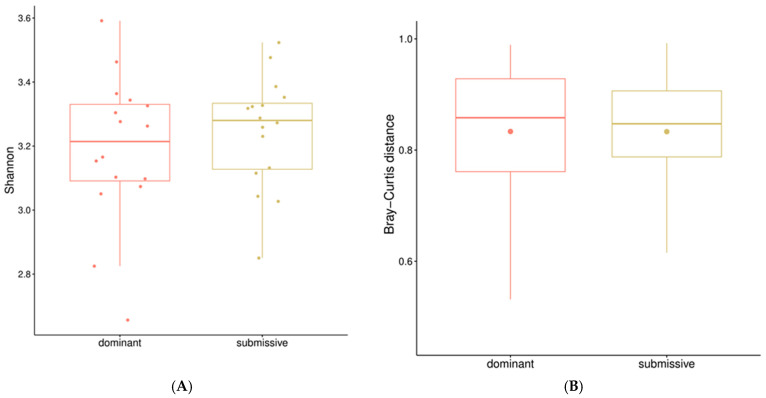
Alpha and beta diversity results. (**A**): Box plot of the Shannon diversity index for each hierarchical class. Higher values indicate higher diversity. No significant differences were found. (**B**): Box plot of the Bray–Curtis distance for both hierarchical classes. Values between 0 and 1 indicate varying degrees of dissimilarity. No significant difference was found.

Shannon-alpha diversity was calculated for each sample within both extreme hierarchical classes: dominant and submissive. Alpha diversity helps understand the number of species present within samples. As shown in Figure 6, no significant difference was found between both groups. Similarly, the beta diversity based on the Bray–Curtis dissimilarity index, which takes into account differences between samples, did not show any significant differences between the dominant and submissive pigs.

#### 3.4.2. Hierarchical-Associated Microbial Biomarkers

**Figure 7:**
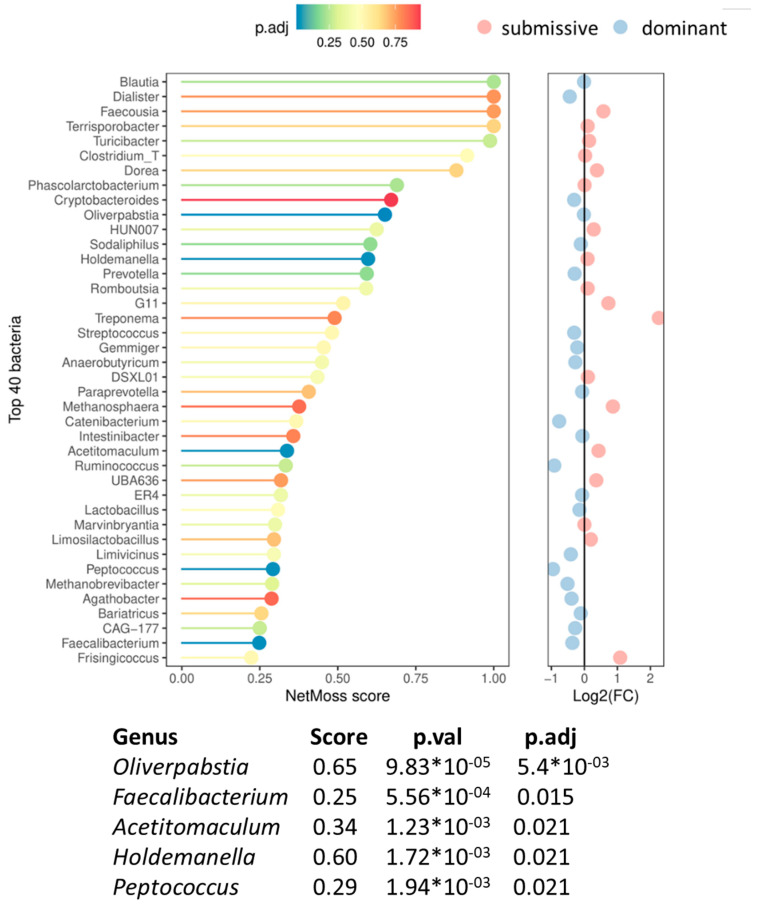
NetMoss results of the differential bacteria between submissive and dominant classes. NetMoss plot showing the top 40 bacteria (vertical axis) with the highest NetMoss score (horizontal axis) in descending order. The colour of each line/bacteria represents the *p*-adjusted value of the difference in abundance between both classes. To the right, the same ranked bacteria are plotted for the logarithm of 2 of the fold-change abundance of the submissive over the dominant. A table of the bacteria found to be significantly (*p* adj. < 0.05) more abundant in either group, sorted by descending order of the NetMoss score, is also shown.

Shifts in microbial network modules were assessed to identify microbial biomarkers associated with hierarchical classes. For this reason, NetMoss2 was employed. Figure 7 represents the genus distribution between class network inference and scoring (score threshold ≥ 0.25). Differential abundance patterns were observed in five genera (*p* adj. < 0.05). Among them, *Oliverpabstia*, *Peptococcus*, and *Faecalibacterium* were significantly more abundant in dominant animals, while *Holdemanella* and *Acetitomaculum* were overrepresented in submissive ones. To be noted, the prosed microbial biomarkers showed a high ability to distinguish between the two experimental conditions (AUC = 0.92).

## 4. Discussion

The present study provides useful data for understanding the feeding behaviour within the hierarchy of a group of pigs and its relationship with the faecal microbiota. The results primarily revealed differences between submissive and dominant animals, with those considered dominant showing greater weight gains at the end of the study. The heavier pigs acting as dominants have already been addressed in the literature [22]. This approach is mainly founded on the premise that larger animals occupy feeders most of the time, resulting in greater consumption and growth [3]. In fact, social hierarchy in pigs can impact growth performance through various mechanisms. A previous study [10] suggested that social ranking influences feeding behaviour, coping styles, and physiological responses, which can indirectly affect growth. Concretely, they found that pigs with dominant status visited the feeder more frequently but spent less time there compared with subordinate and intermediary pigs, which is in accordance with the results found in this study, where more visits were found in dominant than submissive pigs. In this sense, submissive pigs are more affected by competition than dominants, which resembles the results found in previous studies where group-housed pigs were reported to have fewer meals and lower daily feed intake but higher meal size and longer meal duration [23,24], which can restrict growth rate under commercial-like conditions due to feeder competition, aggression, and overall social stress [23,24]. What notably attracts attention and reinforces this rationale are the extreme examples encountered in the observed hierarchical shifts. As the experiment progressed, one animal transitioned from dominance to submissiveness, while another shifted from submissiveness to dominance. These pigs serve as representatives of the behavioural profile of these groups since, as they changed positions in the hierarchy, their feeding behaviours also changed according to what was observed in the hierarchical classes. The connection between hierarchy and weight has also been recently studied through the observation of queue formation at feeders. In non-preferred feeder access locations, the presence of light pigs led researchers to infer that these were submissive animals, while those in better positions in the queue would be dominant [25]. On the other hand, the observed patterns suggest that our proposed dominance test would allow us to identify those animals that would outperform their submissive pen peers in terms of growth, or conversely, those that would underperform in similar circumstances compared with their dominant peers. Therefore, the test is useful for determining the hierarchical position of a pig within a group, particularly if we consider the relationship already discussed between dominance and growth in pigs as a validation source for the model.

Another highly interesting result of the present work is that variance across hierarchy classes was fairly common (only 25% of all animals remained in their original hierarchical class). In addition, no particular hierarchical movement (or lack thereof) was significantly more frequent in any treatment. The only two differences observed were that the most extreme hierarchical changes (dominant to submissive or vice versa) only occurred within the stressed group and that no stressed submissive animal remained submissive for the whole experiment. These results have two consequences. Firstly, in general, the hierarchy in a group of pigs seems to be not very stable along the time, with few differences in animals that are mixed during the growing period in relation to those not mixed. This trend particularly affects intermediate pigs and, to a lesser extent, the dominant ones, who tend to maintain their status more consistently compared with the others. It is important to note that in the present study, the mixing of animals was performed twice—first with females and then with males. The mixing was performed randomly between pens for both dominant and intermediate pigs. However, this was not the case for submissive pigs, as the criterion was to always retain the smallest female and male in their original pen. This approach allowed these potentially last-ranked animals to improve their positions when new animals arrived. This can explain why, in the control group, 25% of the submissive animals remained submissive throughout the study, whereas in the stress group (mixed), none of them remained submissive for the entire study. Therefore, in this case, the model used in the study was helping the submissive animals change their social status.

Another interesting result of the paper was to see how submissive pigs were eating in the central part of the day during the study, carried out mainly in the summer in a warm country such as Spain. In fact, in this case, submissive animals could access food during a high-temperature range, probably because dominants were resting. However, it is important to note that the effect was not visible in the warmest phase of the study (phase 2), although the size of the animals, being the maximum at the latest phase of the study, could explain why the effect was seen at the end of the growing period and not in previous ones. In general, during the summer, pigs prefer to feed early in the morning and early in the evening when the temperature is not as high [26]. In another study looking at the behavioural plasticity of subordinate penmates under limited feeder access (single feeder), the smallest pigs (i.e., outcompeted) ate most of their daily feed intake during the nighttime [3,27]. In general, the literature suggests that pigs can adjust their feeding behaviour in response to constraints within their environment, such as limited access to feeders or social dynamics, to meet their metabolic needs [24].

The faecal microbiota analysis suggests microbial signatures with strong classification capacity (AUC = 0.92) between dominant and submissive pigs, characterised by a higher abundance of beneficial genera *Faecalibacterium* and *Peptococcus* and a lower prevalence of *Holdemanella* in the microbial ecosystems of dominant pigs.

*Faecalibacterium*, a strictly anaerobic butyrate-producing bacterium, is positively associated with human health [28]. In pigs, similar to humans, the relative abundance of *Faecalibacterium* increases with age [29]. The beneficial effects of *Faecalibacterium* in pigs have been well documented, including its link to feeding efficiency [30], reduced incidence of diarrhoea, and increased weight gain in pre-weaned dairy heifers [31]. The presence of *Faecalibacterium* has also been observed to decrease in pigs in a model of social stress [32], suggesting a role in overall health. The production of butyrate by *Faecalibacterium* is particularly important, as butyrate serves as an energy source for colonocytes [33], modulates the immune response [34], and has anti-inflammatory properties [35]. These attributes may contribute to the overall well-being and dominance status of pigs, enhancing their competitive edge in hierarchical structures.

*Peptococcus*, another indicator of dominance, has been reported to be positively associated with body weight, average daily gain, and immunocompetence in pigs [36,37]. The role of *Peptococcus* in promoting growth and immune function suggests that it may contribute to the competitive advantage observed in dominant pigs. The presence of Peptococcus could enhance nutrient absorption and immune responses, leading to better health outcomes and improved growth performance, which might be crucial for maintaining a dominant position within the social hierarchy of pigs.

*Oliverpabstia*, a genus with only one described species, has been related to dominant pigs and has been reported to have increased relative abundance in pigs after weaning [38]. The increase in *Oliverpabstia* post-weaning may be linked to the dietary transitions that occur during this period. This increase could impact gut microbiota composition, influencing metabolic processes and immune responses that favour dominance behaviour. The ability of *Oliverpabstia* to adapt to dietary changes and potentially contribute to metabolic efficiency may provide dominant pigs with a physiological advantage in resource utilisation and growth, further reinforcing their status in the dominance hierarchy.

Meanwhile, *Holdemanella*, a member of the Erysipelotrichaceae family that was most abundant in submissive pigs, has been recently suggested as an indicator of prolonged stress in pigs [39]. Stress is known to alter gut microbiota composition [40], and the prevalence of *Holdemanella* in stressed pigs suggests its involvement in the gut-brain axis. The association of *Holdemanella* with neurological disorders [41], such as Alzheimer’s disease [42], autism spectrum disorder [43], and diabetic cognitive impairment [44], indicates that it may play a role in systemic and neuroinflammation. Understanding how *Holdemanella* contributes to these conditions could provide insights into the microbiota’s role in stress and neurological health. The higher abundance of *Holdemanella* in submissive pigs might reflect their chronic stress and subordinate status within the social hierarchy, potentially exacerbating their health issues and perpetuating their submissive behaviour.

Additionally, *Holdemanella* has been reported to have increased abundance in other diseases such as liver fibrosis [45] and has been suggested as an indicator of the progression of chronic kidney disease [46]. These chronic conditions could further compromise the health and well-being of submissive pigs, making them less competitive and reinforcing their lower status within the dominance hierarchy. The association of *Holdemanella* with these diseases suggests that the microbiota not only reflects but also potentially influences the health disparities between dominant and submissive pigs. This underscores the importance of gut microbiota composition in mediating the physiological and behavioural outcomes linked to social hierarchies in pigs. It is also important to consider the influence of hormonal factors on these behaviours. Due to the use of castrated piglets in the study, it is important to note that the animals never reached the levels of testosterone expected in entire boars, especially at the end of the study, when they would be closer to sexual maturity. Therefore, in other studies using entire males, likely separated from females to avoid unwanted pregnancies, more aggressive and dominant behaviours within the group of males could be expected. Further studies on the effect of the gradual and non-uniform appearance of testosterone in these entire males and its impact on the gut-brain axis could be of great interest to complete the picture found in the present study.

## 5. Conclusions

The present study tested a hierarchical test in pigs that demonstrated a high correlation with the body weight of animals, as it is described in the literature for the relationship between weight and hierarchy in pigs. According to the results, the dominant animals had higher gains during the growing period than the submissive animals. In addition, they were performing more visits to the feeder throughout the day. In contrast, the submissive pigs performed more visits to the feeder at midday, when in a summer environment, as was the case in the study, it is expected that there will be a reduction in the number of visits to the feeder. By means of a repetition of the hierarchy test during the growing period, it was observed how most pigs changed their category (dominant, submissive, or intermediate), although the dominant animals were more stable during the study. When the faecal microbiota was assessed, differential abundance patterns were observed in five genera, showing strong classification capacity. *Oliverpabstia*, *Peptococcus*, and *Faecalibacterium* were more abundant in dominant animals, while *Holdemanella* and *Acetitomaculum* were overrepresented in submissive ones at the end of the study. Therefore, dominant and subordinate animals not only show differences in performance and feeding behaviour but also in some specific microbial biomarkers that can be found in faeces and that can be used to assess the social status of an animal and other aspects related to pig welfare in the future.

## Figures and Tables

**Table 1:** Comparison of Parameters Between Dominant and Submissive Pigs. This table summarizes the observed differences in weight, food consumption, feeding behaviors, and microbial biomarkers between dominant and submissive pigs across different phases of the study. It includes the mean values (with standard deviations) for each group, the nature of the difference, the *p*-value indicating the statistical significance, and whether the difference is considered significant.

Parameter	Phase	Dominant Pigs	Submissive Pigs	Difference	*p*-Value	Significance
Weight (kg)	Phase 3	126 (±2.65)	120 (±2.54)	Greater weight in dominants	0.018	Significant
Food Consumption (g/day)	Phase 2	2745 (±118)	2476 (±97.7)	Higher consumption in dominants	0.049	Significant
Food Consumption (relative)	Phase 2	109% (±4.6%)	97.6% (±2.35%)	Higher consumption in dominants compared with peers	0.034	Significant
Food Consumption (g/day)	Phase 3	3387 (±93.6)	2870 (±104)	Higher consumption in dominants	0.002	Significant
Food Consumption (relative)	Phase 3	107% (±2.85%)	90.9% (±3.27%)	Higher consumption in dominants compared with peers	0.002	Significant
Median Time per Meal (seconds)	Phase 3	897 (±76.0)	955 (±71.2)	More time per meal in submissives	0.427	Not Significant
Frequency of Visits to Feeder	Phase 3	117% (±8.57%)	95.1% (±4.59%)	More visits in dominants compared with peers	0.034	Significant
Daily Feeding Duration	Phase 3	108% (±3.79%)	95.3% (±4.16%)	More total time spent feeding by dominants compared with peers	0.041	Significant
Feeding Time (10:00 h–13:59 h)	Phase 3	20% (±1.10%)	24.1% (±1.26%)	More time spent proportionally by submissives	0.033	Significant
Relative Feeding Time (10:00 h–13:59 h)	Phase 3	93.2% (±4.87%)	110% (±5.49%)	More time spent proportionally by submissives compared with peers	0.030	Significant
Hierarchical Changes	Overall	See text	See text	No significant difference	>0.05	Not Significant
Microbial Biomarkers (Selected Genera)	Phase 3	Oliverpabstia, Peptococcus, Faecalibacterium	Holdemanella, Acetitomaculum	Differential abundance observed	<0.05	Significant
**Parameter**	**Phase Transition**	**Better**	**Worse**	**Difference**	***p*-Value**	**Significance**
Total Daily Intake (grams)	Phase 2 to Phase 3	Reference	−315.78 g	Negative change in hierarchy showed decreased total daily intake	0.034	Significant
Relative Feed Rate	Phase 1 to Phase 3	Reference	−17%	Negative change in hierarchy showed decreased relative feed rate	0.047	Significant

## Data Availability

Data will be available upon request to the corresponding author.
